# Transcriptome-Wide Identification of TCP Transcription Factor Family Members in *Pinus massoniana* and Their Expression in Regulation of Development and in Response to Stress

**DOI:** 10.3390/ijms242115938

**Published:** 2023-11-03

**Authors:** Mengyang Zhang, Romaric Hippolyte Agassin, Zichen Huang, Dengbao Wang, Sheng Yao, Kongshu Ji

**Affiliations:** State Key Laboratory of Tree Genetics and Breeding, Key Open Laboratory of Forest Genetics and Gene Engineering of National Forestry and Grassland Administration, Co-Innovation Center for Sustainable Forestry in Southern China, Nanjing Forestry University, Nanjing 210037, China

**Keywords:** *Pinus massoniana*, TCP, stress response, hormone, development, expression pattern

## Abstract

*Pinus massoniana* is an important coniferous tree species for barren mountain afforestation with enormous ecological and economic significance. It has strong adaptability to the environment. TEOSINTE BRANCHED 1/CYCLOIDEA/PCF (TCP) transcription factors (TFs) play crucial roles in plant stress response, hormone signal transduction, and development processes. At present, TCP TFs have been widely studied in multiple plant species, but research in *P. massoniana* has not been carried out. In this study, 13 PmTCP TFs were identified from the transcriptomes of *P. massoniana*. The phylogenetic results revealed that these PmTCP members were divided into two categories: Class I and Class II. Each PmTCP TF contained a conserved TCP domain, and the conserved motif types and numbers were similar in the same subgroup. According to the transcriptional profiling analysis under drought stress conditions, it was found that seven *PmTCP* genes responded to drought treatment to varying degrees. The qRT-PCR results showed that the majority of *PmTCP* genes were significantly expressed in the needles and may play a role in the developmental stage. Meanwhile, the *PmTCPs* could respond to several stresses and hormone treatments at different levels, which may be important for stress resistance. In addition, PmTCP7 and PmTCP12 were nuclear localization proteins, and PmTCP7 was a transcriptional suppressor. These results will help to explore the regulatory factors related to the growth and development of *P. massoniana*, enhance its stress resistance, and lay the foundation for further exploration of the physiological effects on PmTCPs.

## 1. Introduction

Transcriptional regulation is an important mechanism for regulating gene expression during plant growth, development, and metabolism [1]. Transcription factors (TFs) are regulatory proteins that can directly or indirectly interact with *cis*-acting elements in promoters to regulate the expression of target genes, thereby regulating plant development and responding to external stresses [2]. Previous studies have analyzed the conserved regions of TEOSINTE BRANCHED 1 (TB1) protein of *Zea mays*, CYCLOIDEA (CYC) protein of *Antirrhinum majus*, and PROLIFERATING CELL TRANSCRIPTION FACTORS 1/2 (PCF1/2) of *Oryza sativa.* It was found that they all contained the bHLH conserved domain; therefore, the TEOSINTE BRANCHED 1/CYCLOIDEA/PCF (TCP) TF family was defined [3]. Presently, TCP TFs have been studied in many species, for example, *Arabidopsis thaliana* [4], *Gossypium raimondii* [5], and *Malus domestica* [6].

The structural characteristics of TCP TFs are that they have a basic Helix–Loop–Helix (bHLH) structure, also known as the TCP domain, which mainly participates in protein interactions and DNA binding [7,8]. TCP TFs can be divided into two categories: Class I and Class II. The Class II protein members have four more conserved amino acid residues than Class I protein members in the basic region of the TCP domain [9]. Class I is also named the PCF subfamily. According to the differences in amino acid sequences, especially the differences in the basic regions, the Class II members can be further divided into CYC/TB1 and CIN subfamilies. In addition, a few members of the CIN subfamily contain an arginine-rich motif (R domain), while the majority of members of the CYC/TB1 subfamily contain the R domain [10,11]. The members of Class I can promote cell differentiation and plant growth [12]. CYC/TB1 subfamily (Class II) members influence the symmetry of plant floral patterns and inhibit the growth of axillary buds, thereby reducing the number of branches [13,14]. The members of the CIN subfamily (Class II) can regulate leaf development [4].

TCP TFs play important roles in various biological functions of plants, including seed germination, organ differentiation, hormone regulation, signal transduction, and stress response [15,16,17]. Relevant studies have shown that *AtTCP4* can promote the biosynthesis of auxin by regulating the expression of genes related to auxin biosynthesis enzymes [18]. *AtTCP4* can induce the expression of lipoxygenase-related genes, which promotes the synthesis of methyl jasmonate (MeJA) and the senescence of mature leaves [19]. *PeTCP10* responds to abscisic acid (ABA) signals and enhances tolerance to drought stress [20]. *OsPCF6* and *OsTCP21* are target genes of microRNA319, which can significantly improve plant resistance to low temperatures [21]. The *AtTCP14* gene can directly activate the *cis*-acting elements in vascular tissue that regulate axillary bud growth, thus mediating the expression of germination-related genes and promoting plant growth [22]. After *Ziziphus jujuba* is infected with phytoplasma, the expression levels of *ZjTCP* genes increase in leaves and phloem, indicating that *ZjTCPs* have important roles in resistance to phytoplasma [23].

*Pinus massoniana* is the pioneer tree of afforestation in barren mountains in south China. It is the main coniferous tree species and is generally used in construction, timber, and papermaking; it also plays crucial roles in environmental regulation and increasing carbon sequestration [24]. *P. massoniana* can secrete pine resin, which is a secondary metabolite mainly composed of terpenoids. Its components have significant functions on the defense systems [25,26,27]. However, *P. massoniana* is always affected by various unfavorable factors throughout its entire growth process. The distribution environment of *P. massoniana* is complex, and the uneven distribution of rainfall limits the growth of *P. massoniana* and limits its expansion in area [28]. Drought stress leads to stomatal closure of *P. massoniana*, which seriously reduces its photosynthetic activity [29]. To adjust to complex and changeable conditions, plants have evolved various regulatory mechanisms. Moreover, TCP TFs play crucial roles in numerous biological processes in plants [30,31]. For example, the *AtTCP10* gene is involved in cell differentiation, leaf morphogenesis, and other biological processes [32]. Under bright light conditions, *AtTCP15* can regulate anthocyanin accumulation and protect plant cells from damage [33]. Therefore, it is of great significance to study the functions of TCP TFs of *P. massoniana*. At present, there are still no available genome resources for *P. massoniana*. Therefore, we identified the members of the PmTCP TF family based on the transcriptomes, namely, CO_2_ stress transcriptome, tender shoots transcriptome, and drought stress transcriptome. Furthermore, identification and analysis of 13 PmTCP TFs using bioinformatics methods. In addition, *PmTCP* genes were used for expression pattern analysis under different conditions. These results not only laid a theoretical foundation for the study of PmTCP TFs but also provided a reference for the response mechanism to external stress of *P. massoniana*.

## 2. Results

### 2.1. Identification of TCP Family Proteins in P. massoniana

A total of 13 TCP proteins were identified from the *P. massoniana* transcriptomes after removing sequences without conserved structural domain and duplicate sequences. Then, the 13 PmTCP TFs were named PmTCP1 to PmTCP13, and their protein sequences were used for physicochemical properties analysis (Table S1). These protein sequences varied from 182 (PmTCP4) to 626 (PmTCP10) amino acids (aa) in length, with the molecular weight (MW) varying from 19,932.49 (PmTCP4) to 68,110.09 (PmTCP10) Da. The isoelectric point (pI) values ranged from 6.69 (PmTCP2) to 9.49 (PmTCP7), and the aliphatic index varied from 52.27 (PmTCP11) to 74.07 (PmTCP4). In addition, the instability index (II) of PmTCP TFs was all higher than 40, indicating that they were unstable proteins. The grand average of hydropathicity (GRAVY) values were all negative, indicating that PmTCPs were hydrophilic proteins.

### 2.2. Phylogenetic Analysis of the TCP Proteins

**Figure 1:**
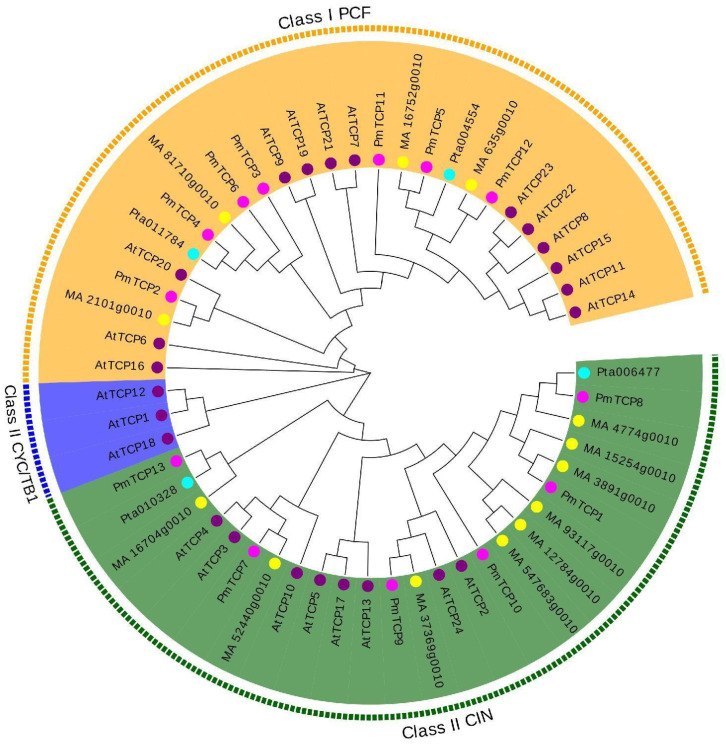
Phylogenetic tree of the TCP protein family members of *P. massoniana*, *A. thaliana*, *P. abies*, and *P. taeda*. The different color backgrounds and surrounding letters denote different groups. The magenta circles, purple circles, yellow circles, and cyan circles represent TCP TFs of *P. massoniana*, *A. thaliana*, *P. abies*, and *P. taeda*, respectively.

A phylogenetic evolutionary tree was constructed comprising 13 TCP protein sequences from *P. massoniana*, 24 TCP protein sequences from *A. thaliana*, 13 TCP protein sequences from *Picea abies*, and 4 TCP protein sequences from *Pinus taeda*. The clustering results (Figure 1) showed that all TCP TFs were classified into Class I PCF, Class II CIN, and Class II CYC/TB1. According to the evolutionary classification of TCP TFs in *A. thaliana*, the TCP TFs of the species were divided into two groups, namely, Class I and Class II. Class I—also known as the PCF subfamily—and Class II could be further divided into CYC/TB1 and CIN subfamilies (Figure 1). 7 PmTCP TF members were distributed in the PCF subfamily. The CIN subfamily contained six PmTCP TF members. In addition, *Arabidopsis* TCP members were observed in all subfamilies, while three gymnospermic TCP members were only found in PCF and CIN subfamilies, indicating that TCP TFs of *P. massoniana* were more closely related to *P. abies* and *P. taeda* in a phylogenetic relationship.

### 2.3. Conserved Structural Domain Analysis of PmTCP TFs

**Figure 2:**
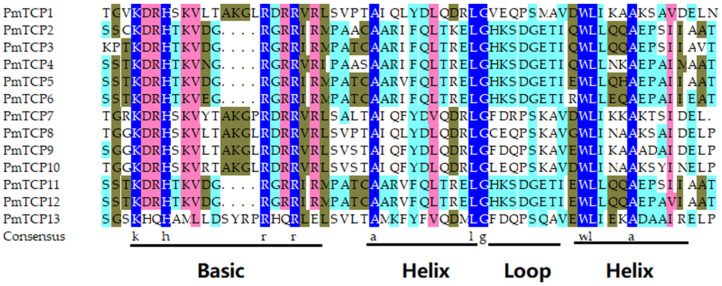
Multiple sequence alignments of conserved domains of 13 TCP family proteins in *P. massoniana*. The brown, light red, light blue, and dark blue backgrounds imply protein identities of 33%, 50%, 75%, and 100%, respectively. The HLH region and basic region are marked with black lines.

To confirm the conserved domain of the PmTCP TFs, multiple sequence alignments of conserved domains were performed using DNAMAN 6.0 software. The result is shown in Figure 2. All PmTCP proteins contained a complete TCP domain, including a Helix–Loop–Helix domain and a basic domain. In the basic domain, Class I members PmTCP2, PmTCP3, PmTCP4, PmTCP5, PmTCP6, PmTCP11, and PmTCP12 lacked four amino acid residues compared to Class II members. In the HLH region, conservative characteristic sites were also present with lines. The results indicated that PmTCP proteins were highly conserved.

### 2.4. Conserved Motif Identification of PmTCP TFs

**Figure 3:**
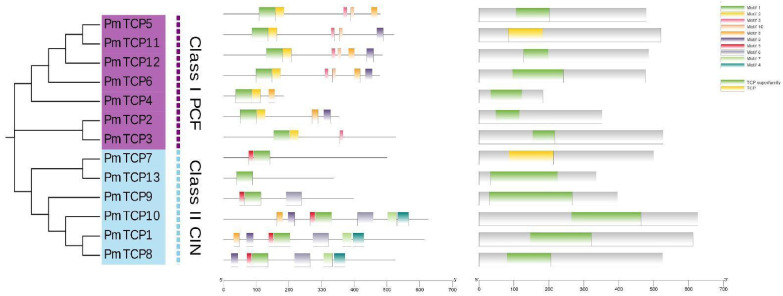
Phylogenetic tree, Conserved motif distribution, and structural domain analysis of the TCP proteins in *P. massoniana*. The motif structures were acquired by MEME analysis. Ten conserved motifs are presented of PmTCP TFs, and the different colors indicate different kinds of motifs.

A total of 10 conserved motifs were determined in the 13 PmTCP family TFs by the MEME 5.5.4 software, and they were named motifs 1–10. The sequences of motifs are listed in Table S2. The number of amino acids in the 10 motifs ranged from 11 to 50, and motif 1 was the characteristic domain of TCP TFs. The distribution pattern of the motifs is shown in Figure 3. All PmTCP proteins contain the conserved motif 1, indicating that the structures of PmTCP TFs were complete. In addition, members of the same subfamily generally contained similar motif types. For instance, all members of Class I contained motifs 1 and 2. In addition, there were some differences in the motif types of different subfamilies. For example, motifs 2, 3, and 10 only exist in Class I members; motifs 5, 7, and 4 were only found in Class II members. The results suggested that the distribution of motifs was consistent with the evolutionary classification, and the differences in motifs of different members may imply the differences in protein structure.

### 2.5. Subcellular Localization of PmTCP TFs

**Figure 4:**
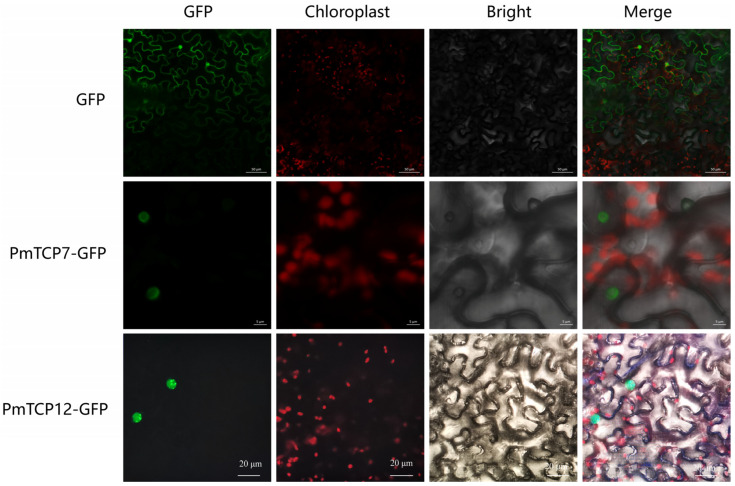
Subcellular localization experiments of PmTCP7 and PmTCP12 proteins. Transient expression of GFP (control), PmTCP7-GFP, and PmTCP12-GFP in tobacco leaves. The scale bar in the images of GFP is 50 μM, the scale bar in the images of PmTCP7-GFP is 5 μM, and the scale bar in the images of PmTCP12-GFP is 20 μM.

The Cell-PLoc 2.0 software predicted that all PmTCP TFs were nuclear localization proteins (Table S3). To further verify the subcellular localization characteristics of PmTCP TFs, PmTCP7, and PmTCP12 were randomly selected from the Class II (CIN) and Class I (PCF) subfamilies for instantaneous transformation experiments, respectively. They were highly expressed in the drought-stressed transcriptome. The fluorescence signal was observed in the instantaneously transformed tobacco leaves (Figure 4). The GFP signal was found to be distributed throughout the whole cell in the control; however, GFP fused with PmTCP7 and PmTCP12 only observed fluorescence signals in the nucleus. The results showed that PmTCP proteins were localized in the nucleus.

### 2.6. Transcriptional Analysis of PmTCPs Using Drought Transcriptome Data

**Figure 5:**
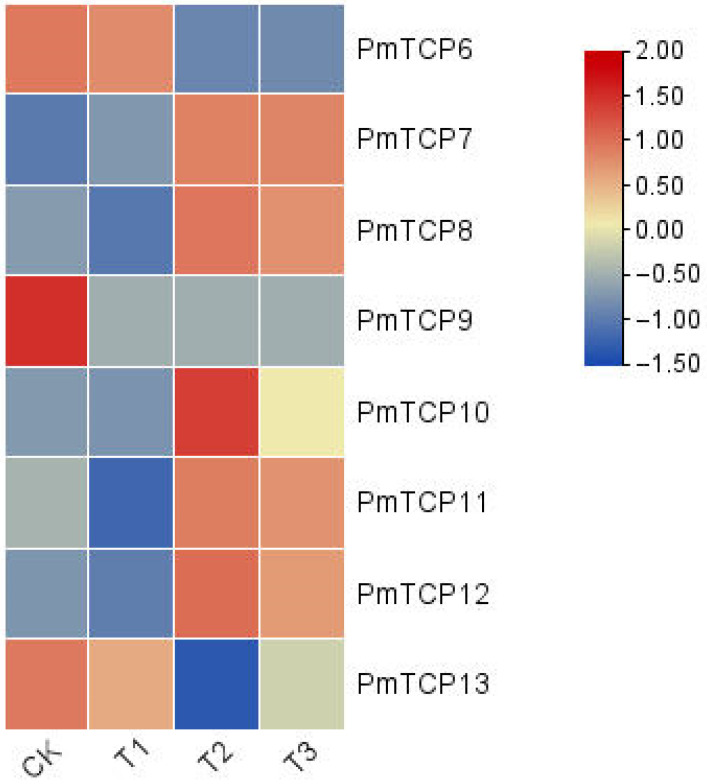
Transcriptional analysis of *PmTCP* genes in *P. massoniana* under four drought stresses: CK (80 ± 5)%, T1 (65 ± 5)%, T2 (50 ± 5)%, and T3 (35 ± 5)%. A heatmap was generated using the log_2_(FPKM+1) value, and the color scale denotes the relative expression level.

To study the potential function of *PmTCP* genes in drought stress, the heatmap of *PmTCP* genes was constructed based on the drought stress transcriptome data (PRJNA595650). Under drought stress conditions, the expressions of several genes were too low to be detected, so only the expression levels of *PmTCP6–PmTCP13* genes were given (Figure 5). The results demonstrated that the expression patterns of *PmTCP* genes were different. For example, the expression level of the *PmTCP7* gene increased whether it was mild drought, moderate drought, or severe drought. The expression patterns of *PmTCP8*, *PmTCP11*, and *PmTCP12* genes were similar; their expression levels slightly decreased under mild drought but increased under moderate and severe drought. The expression of *PmTCP10* only significantly increased under moderate drought conditions. However, the expression levels of *PmTCP6* and *PmTCP13* genes were significantly down-regulated after moderate and severe drought treatment. *PmTCP9* expression significantly decreased throughout the drought treatment. These results indicated that *PmTCP* genes could be responsive to drought and may play vital roles in drought conditions.

### 2.7. Expression Patterns of PmTCPs in Various Tissues

**Figure 6:**
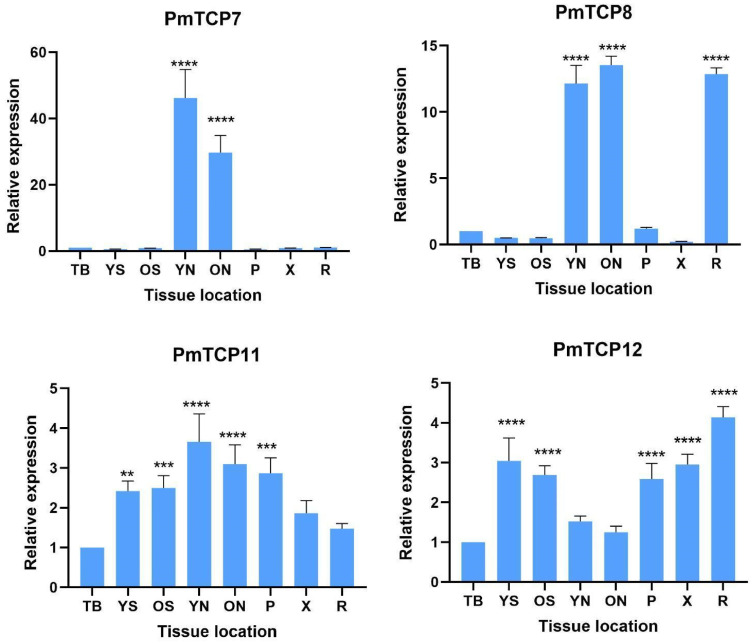
The expression levels of *PmTCPs* in eight different tissues in *P. massoniana*. TB: terminal bud; YS: young stems; OS: old stems; YN: young needles; ON: old needles; P: phloem; X: xylem; R: roots. The relative expression level is indicated as the mean ± standard deviation (SD). Asterisks show significant differences in the expression level between the treated groups and the control group (0 h) (** *p <* 0.01, *** *p <* 0.001, **** *p <* 0.0001).

The expression levels of four selected *PmTCP* (*PmTCP7*, *PmTCP8*, *PmTCP11*, *PmTCP12*) genes were analyzed by the quantitative reverse transcription *PCR* (qRT-PCR) in eight tissues: terminal bud (TB), young stems (YS), old stems (OS), young needles (YN), old needles (ON), phloem (P), xylem (X), and roots (R). These genes were selected based on their high expression levels in the drought-stressed transcriptome. The results (Figure 6) indicated that *PmTCP* genes were expressed differently in these eight tissues. *PmTCP7* was found to be expressed at higher levels in young and old needles than in other tissues. The expression levels in young needles and old needles were 46.2 and 29.7 times those in terminal buds, respectively. The expression of *PmTCP8* in young needles, old needles, and roots was significantly higher than that in other tissues, which was 12.2, 13.5, and 12.9 times that in terminal buds, respectively. *PmTCP11* was also highly expressed in young and old needles; its expression level was 3.1–3.7 times that of the terminal buds. The expression levels in young stems, old stems, and phloem were approximately 2.4–2.9 times that in terminal buds. *PmTCP12* had the highest expression level in roots, approximately 4.1-fold of that in terminal bud. The expression levels in young stems, old stems, phloem, and xylem were about 2.4, 2.7, 2.6, and 3.0 times those in terminal buds, respectively. The expression level of *PmTCP12* in young needles and old needles was relatively consistent with those in terminal buds.

### 2.8. Expression Levels of PmTCP Genes Respond to Different Treatments

**Figure 7:**
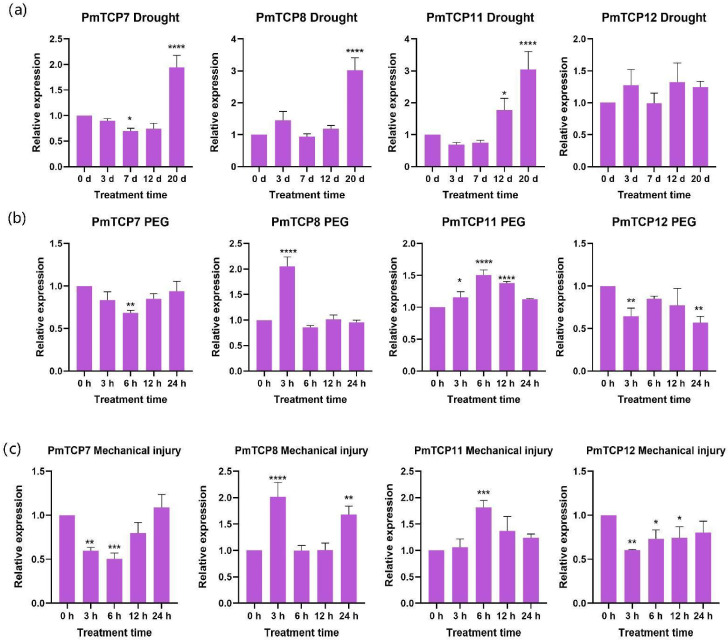

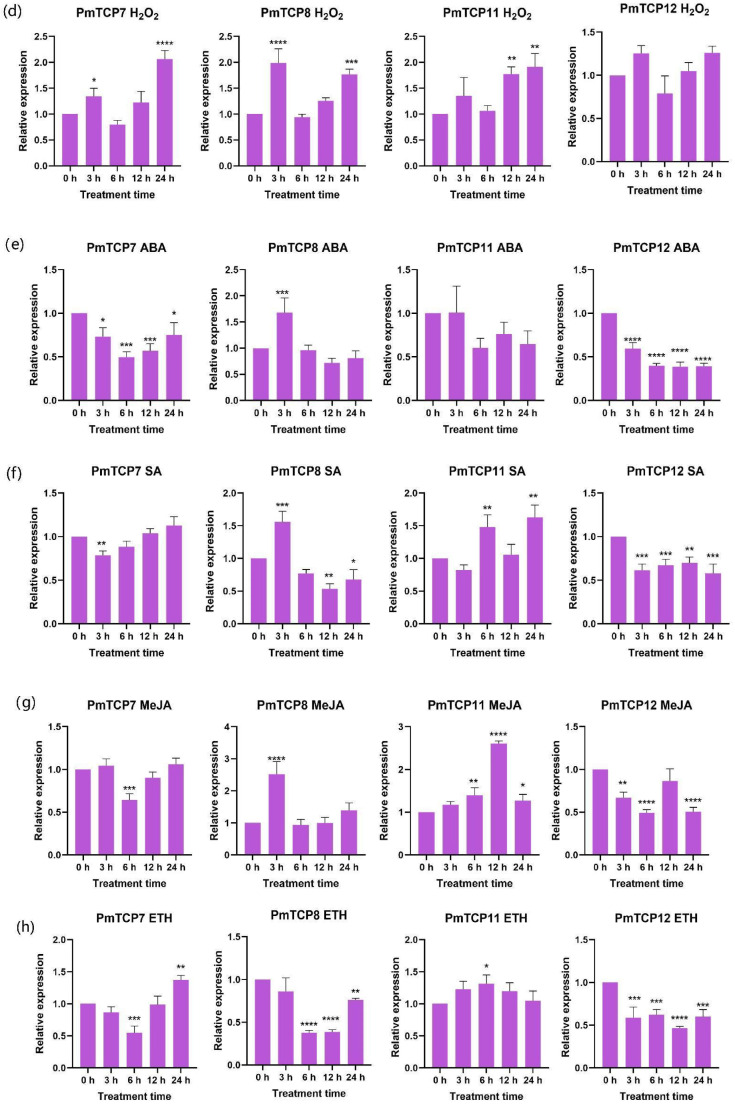
The expression patterns of *PmTCP* genes under different treatments. (**a**) drought; (**b**) PEG; (**c**) mechanical injury; (**d**) H_2_O_2_; (**e**) ABA; (**f**) SA; (**g**) MeJA; (**h**) ETH. The relative expression is indicated as the mean ± standard deviation (SD). Asterisks show significant differences in the expression level between the treated groups and the control group (0 h) (* *p* < 0.05, ** *p* < 0.01, *** *p* < 0.001, **** *p* < 0.0001).

The expression patterns of these four *PmTCP* genes under eight distinct treatments are presented in Figure 7. Under drought conditions, the expression of *PmTCP7* and *PmTCP8* significantly increased at 20 d. *PmTCP11* expression increased at 12 d and 20 d and reached the highest expression level at 20 d. There was no significant difference in the expression of *PmTCP12* throughout the process. Under PEG treatment conditions, the expression of *PmTCP7* and *PmTCP12* slightly decreased during the process. The expression level of *PmTCP8* increased at 3 h and then decreased. The expression level of *PmTCP11* also increased throughout the process. Under mechanical injury treatment, the expression level of *PmTCP7* decreased at 3 h and 6 h, indicating that the expression of *PmTCP7* was inhibited under mechanical injury treatment. The expression of *PmTCP8* was upregulated at 3 h and 24 h. *PmTCP11* reached the highest expression level at 6 h. *PmTCP12* expression slightly decreased. After SA treatment, the expression level of *PmTCP7* slightly decreased at 3 h. *PmTCP8* expression increased at 3 h and then showed a decreased trend. The expression profile of *PmTCP11* was higher than that of the control at 6 h and 24 h. The expression level of *PmTCP12* decreased during the process. Under MeJA treatment, the expression of *PmTCP7* decreased at 6 h, and the results showed that *PmTCP7* expression was suppressed at a certain time point during MeJA treatment. *PmTCP8* and *PmTCP11* had the highest expression levels at 3 h and 12 h, respectively. The expression of *PmTCP12* was downregulated. After ETH treatment, the expression pattern of *PmTCP7* was lower than that of control at 6 h and reached its peak at 24 h. The expression levels of *PmTCP8* and *PmTCP12* were downregulated throughout the process. *PmTCP11* expression pattern tended to increase slightly at 6 h. Under H_2_O_2_ treatment, the expression trends of *PmTCP7* and *PmTCP8* first increased, subsequently decreased, and then increased. The expression of *PmTCP11* was upregulated at 12 h and 24 h. *PmTCP12* expression pattern tends to stabilize, and the response to H_2_O_2_ was not significant. Under ABA treatment, the expression levels of *PmTCP7* and *PmTCP12* were downregulated and suppressed throughout the ABA process. The expression of *PmTCP8* increased at 3 h. There was no significant difference in the expression of *PmTCP11* throughout the whole process.

### 2.9. Transcriptional Activity Analysis of PmTCP7

**Figure 8:**
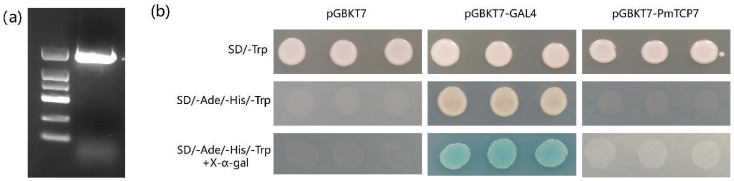
Transcriptional activity assay of PmTCP7. (**a**) PCR amplification of *PmTCP7* gene; (**b**) Transcriptional activity analysis, pGBKT7, was used as a negative control, and pGBKT7-GAL4 was used as a positive control.

Moreover, transcriptional activity of PmTCP7 was detected and analyzed (Figure 8). The *PmTCP7* gene was successfully cloned. This gene had the highest expression in the drought-stressed transcriptome and responded to several hormonal treatments. In addition, its expression level is high in tissues, especially in needles. Yeast cells carrying the fusion expression vector of pGBKT7-PmTCP7 could grow on SD/-Trp yeast medium; however, it was unable to grow on yeast SD/-Trp/-Ade/-His selective media and did not display blue spots on SD/-Trp/-Ade/-His yeast media containing X-α-gal. This is depicted in various graphics. The findings indicated that PmTCP7 was a transcriptional inhibitor; this study laid the groundwork for further research on gene regulation.

## 3. Discussion

TCP TFs have been identified in many species, for example, 24 TCP TFs in *A. thaliana* [4], 22 TCP TFs in *O. sativa* [4], 33 TCP TFs in *Populus euphratica* [34], 9 TCP TF members in pineapple [35], and 18 VvTCPs were identified in *Vitis vinifera* [36]. The TCP family is widely involved in multiple biological processes in plants that have important effects on regulating plant growth, defense responses, and hormone signal transduction. Increasingly, studies have shown that TCP genes are promising candidates with regard to plant resistance and development [37,38,39]. In addition, *P. massoniana* has strong adaptability to the environment. Therefore, reports on the correlation between TCP TFs and *P. massoniana* stress and growth are meaningful. Therefore, this study provides a comprehensive analysis of the TCP TF members of *P. massoniana,* which combines PmTCPs with the aspects related to growth and stress, providing a basis for further understanding the biological functions of PmTCP TFs.

Thirteen PmTCP TF members were identified from the transcriptomes of *P. massoniana* and named PmTCP1–PmTCP13. According to the classification of *A. thaliana* TCP TFs, The 13 PmTCP members were divided into Class I and Class II, where Class I contained 7 PmTCP proteins and Class II contained 6 PmTCP TF members, with a ratio of about 1:1. Relevant studies have shown that there were 10 VvTCP TFs in Class I and 8 VvTCPs in Class II of *V. vinifera* [36]. In *Prunus mume*, Class I contained 10 TCP members, and Class II contained 9 TCP members [40], which was also close to 1:1. These results were consistent with the current results. Multiple sequence alignment analysis showed that these PmTCP TFs had bHLH functional conserved domains. The conserved domains of Class I proteins lacked four amino acid residues compared to Class II proteins, which may lead to differences in function. This phenomenon has also been observed in tobacco [41]. Motif 1, a characteristic motif of TCP protein, exists in all PmTCP amino acid sequences and is an important basis for identifying TCP members. In the same subgroup members, the number and type of conserved motifs were similar, and the distribution order was the same. However, different subgroup members had their own representative motifs, which strongly supported the phylogenetic relationship and classification of PmTCPs. The difference and consistency of motif distribution provided a foundation for the functional diversity and consistency of PmTCP TFs. This pattern was also observed in *Cucumis sativus* [42]. Overall, these results suggested that PmTCP TFs were structurally conservative.

The results of tobacco transient expression experiments and subcellular localization prediction implied that PmTCP TFs are nuclear localization proteins, which is consistent with the localization characteristics of TFs and the theory that TFs normally play roles in the nucleus.

To some extent, the expression patterns of genes are related to their functions. It has been reported that TCP TFs are involved in many aspects of plant biological processes. Drought resistance is an important response mechanism for plants to adapt to external stresses [28]. Therefore, the expression profile of PmTCP TFs was analyzed using the drought stress transcriptome data of *P. massoniana*. It was found that the expression levels of *PmTCP7*, *PmTCP8*, *PmTCP11*, and *PmTCP12* genes significantly increased after moderate and severe drought treatments. The expression of the *PmTCP10* gene was significantly upregulated after moderate drought stress. The expressions of *PmTCP6*, *PmTCP9,* and *PmTCP13* genes were significantly downregulated after drought stress treatment. Additionally, there was no significant difference in the expression of most *PmTCP* genes compared to the control under mild drought, while the expression levels were obviously increased or decreased under moderate and severe drought conditions. It is speculated that *PmTCPs* could be responsive to drought stress, but their response mechanisms were complex and different. These findings laid a foundation for further research on the function of *PmTCP* genes in drought stress.

It has been observed that the function of the *TCP* genes can regulate the growth process of many tissues in plants, such as stems, leaves, flowers, and roots [32,43]. To investigate the expression characteristics of *PmTCP* genes in tissues, the expression of *PmTCP* members in different tissues of *P. massoniana* was analyzed. The results indicated that *PmTCP* genes could be expressed in various tissues. The expression levels of *PmTCP7* and *PmTCP8* genes in young needles and old needles were significantly higher than those in other tissues; in addition, *PmTCP8* was also highly expressed in roots. According to the phylogenetic evolutionary relationships, *PmTCP7* and *PmTCP8* belong to Class II members. It has been reported that Class II members are specifically expressed in plant tissues and participate in the formation of leaf trichosomes and roots [44]. These results suggested that *PmTCP7* and *PmTCP8* may have regulation roles in needles and roots.

Relevant studies have shown that TCP TFs can respond to a variety of hormone and abiotic stresses [45,46]. The expression patterns of *PmTCP* genes were determined under different treatments. The expression of *PmTCP7*, *PmTCP8*, and *PmTCP11* significantly increased under drought stress, and the expression of *PmTCP8* and *PmTCP11* was upregulated after PEG treatment. The results indicated that these genes had a positive response to drought and PEG stress, which may induce an increase in POD and SOD enzyme activities, as well as MDA and Pro contents, thereby enhancing osmotic regulation and reducing damage to *P. massoniana* [47]. The increased expression levels of *PmTCP8* and *PmTCP11* under mechanical injury suggested that they may be involved in biological processes, such as preventing the invasion of pathogens. SA, MeJA, ETH, H_2_O_2,_ and ABA are important exogenous exciters that are induced by external stimuli and have an important role in response to stress [48]. Through gene expression levels, it was found that *PmTCP7*, *PmTCP8,* and *PmTCP11* positively responded to H_2_O_2_. Meanwhile, *PmTCP8* and *PmTCP11* had a positive response to MeJA and SA. *PmTCP8* and *PmTCP7* could respond to ABA and ETH, respectively. The results showed that these genes were involved in signal transduction and stress perception, resulting in the adaptation changes of *P. massoniana*. The expression of *TCP* genes has been analyzed in many plants under these treatments; for example, *AtTCP20* is involved in the biosynthesis of jasmonic acid [19]. Overexpression of the *OsTCP19* gene can significantly improve drought resistance in mature plants and seedlings [49]. In general, *TCP* genes are widely involved in stress resistance and hormone signaling pathways in plants. However, further functional research is needed to determine how the *TCP* genes accurately respond to these signaling.

By observing the growth conditions of yeast cells containing pGBKT7–PmTCP7 fusion expression vector on the selected medium, it was impossible to detect the transcriptional self-activation activity of these cells. This study provides a basis for studying the regulatory network associated with TCP proteins.

In summary, this study comprehensively analyzed the TCP TFs family of *P. massoniana*. It not only contributes to screening suitable candidate genes for further investigation but also contributes to understanding the molecular regulatory mechanism and signal transduction of *TCP* genes in plant resistance and development. In the meantime, the functional characterization of *PmTCPs* provides insights into the function of TCP TFs in *P. massoniana*.

## 4. Materials and Methods

### 4.1. Identification and Sequence Analysis of TCP Family Members of P. massoniana

A hidden Markov model (HMM) of the TCP conserved domain (PF03634) was acquired through the Pfam database (http://pfam.xfam.org/ (accessed on 17 April 2023)) [50], and TCP protein members were searched using the HMMER 3.0 software from the *P. massoniana* transcriptomes with its defined threshold of E < 10^−5^. The transcriptomes of *P. massoniana* originated from the previously determined drought stress transcriptome (PRJNA595650), CO_2_ stress transcriptome (PRJNA561037), and tender shoots transcriptome (PRJNA655997). PmTCP proteins were identified from these transcriptomes. The SMART online website (http://smart.embl-heidelberg.de/, accessed on 18 April 2023) was used to predict the conserved domain, and proteins without structural domain were deleted. Then, the protein sequences were aligned, and sequences with similarity exceeding 97% were deleted. The protein sequences are listed in Table S4. The physicochemical properties, such as instability index (II), grand average of hydropathicity (GRAVY), isoelectric point (pI), and molecular weight (MW) of the screened TCP proteins were analyzed with ExPASy (http://web.expasy.org/protparam/ (accessed on 19 April 2023)).

### 4.2. Phylogenetic Evolutionary of TCP TFs

The TCP protein sequences of *A. thaliana*, *P. taeda,* and *P. abies* were downloaded from the Plant Transcription Factor Database (http://planttfdb.cbi.pku.edu.cn/ (accessed on 23 April 2023)). Their protein sequences and those of *P. massoniana* were aligned with the ClustalW 2.1 program. A phylogenetic evolutionary tree was constructed using the Neighbor-Joining (NJ) method with MEGA 7.0 software; the bootstrap value was 1000, and other parameters were default values [51]. Subsequently, the Evolview online tool (http://evolgenius.info//evolview-v2/ (accessed on 27 April 2023)) was used to embellish the phylogenetic tree.

### 4.3. Multiple Sequence Alignments and Motif Analysis

DNAMAN 6.0 software was used to align the protein sequences of PmTCP members and coTBtools their conserved domains. The Multiple Em for Motif Elicitation (MEME) (http://meme.nbcr.net/meme/intro.html (accessed on 30 April 2023)) online website was used to obtain the conserved motifs organization of the 13 PmTCP proteins, and 10 motifs were searched using default parameters [52]. Then, the TBtools-II v2.019 platform was used to analyze their conserved structures and produce diagrams [53].

### 4.4. Subcellular Localization Analysis

The Cell-PLoc 2.0 (http://www.csbio.sjtu.edu.cn/bioinf/Cell-PLoc-2/ (accessed on 1 July 2023)) online software was used to predict subcellular localization of PmTCP proteins. *PmTCP7* and *PmTCP12* were randomly selected for transient transformation experiments. The primers for constructing vectors are shown in Table S5. The open reading frame (ORF) regions of *PmTCP7* and *PmTCP12* without stop codon were linked with pBI121-GFP vector, the 35S::*PmTCP7*-GFP and 35S::*PmTCP12*-GFP expression vectors were constructed by recombinase. Then, the vectors were transferred to *Agrobacterium* GV3101. These strains, along with P19 (RNA Silencing Inhibitor) *Agrobacterium* strains, were cultured in LB media for 48 h at 28 °C. Then, they were suspended in a solution containing 150 μM acetosyringone, 10 mM MgCl_2_, and 10 mM 2-(N-morpholino)ethanesulfonic acid (MES). The suspension cells were mixed with p19 at a ratio of 1:1, and the *Nicotiana benthamiana* leaves were injected with the mixed solution. Subsequently, the infiltrated *N. benthamiana* plants were maintained for 3 d under the conditions of 16 h light and 8 h dark photoperiod. The LSM710 confocal microscope (Zeiss, Jena, Germany) was used to capture the GFP signal.

### 4.5. Transcriptional Profile Analysis of the PmTCP Genes

To study the expression of *PmTCP* genes, we utilized the original drought-stressed transcriptome (PRJNA595650) of *P. massoniana* to obtain the RNA sequencing (RNA-seq) data. The soil moisture content for the growth of *P. massoniana* was set to four gradients: normal (CK), mild (T1), medium (T2), and serious (T3). The corresponding water holding capacity was CK (80 ± 5)%, T1 (65 ± 5)%, T2 (50 ± 5)% and T3 (35 ± 5)%, respectively. They were placed at 75% humidity for 60 d and subsequently sequenced. Fragments per kilobase of exon model per million mapped fragments (FPKM) values (Table S6) were computed to assess the expression level of *PmTCPs*. Then, a heat map was generated based on log_2_(FPKM+1) values using TBtools-II v2.019 software.

### 4.6. Plant Materials and Treatments

Two-year-old *P. massoniana* seedlings were planted in pots containing a soil mixture. The volume ratio of vermiculite, perlite, and peat of the soil mixture was 3:1:1. Then, seedlings were placed under the conditions with a temperature of 24 °C and photoperiod of 16 h light and 8 h dark for growth. When *P. massoniana* seedlings grew for about a month and remained in stable conditions, the seedlings with consistent growth status were selected for subsequent treatments.

To investigate the expression levels of *PmTCP* genes, eight tissues were sampled from the seedlings, including young needles, old needles, young stems, old stems, phloem, xylem, terminal buds, and roots. Furthermore, the seedlings were treated with these treatments: 1 mM salicylic acid (SA), 10 mM methyl jasmonate (MeJA), 50 µM ethephon (ETH), 100 µM abscisic acid (ABA), 10 mM hydrogen peroxide (H_2_O_2_), 15% polyethylene glycol (PEG6000), mechanical injury, and drought. The mechanical injury was carried out by cutting off the upper half of the seedling; 15%PEG treatment involved immersing seedlings in this solution, causing osmotic stress. Drought treatment was performed with watering at 0 d and then naturally evaporated for 20 d. The remaining treatments were all sprayed on the surface of the seedlings. Afterward, samples were collected at 0 h, 3 h, 6 h, 12 h, and 24 h, except for drought. The drought-treated samples were collected at 0 d, 3 d, 7 d, 12 d and 20 d. The expression levels of the untreated samples were used as controls. Three biological replicates were used for each treatment.

### 4.7. RNA Extraction and qRT-PCR Analysis

Total RNA was extracted using a FastPure Plant Total RNA Isolation Kit (RC401-01, Vazyme Biotech, Nanjing, China). Then, a NanoDrop2000 (Thermo Fisher Scientific, Waltham, MA, USA) was used to detect the purity and concentration of RNA. Afterward, 1000 ng total RNA was reverse-transcribed to synthesize 20 μL of cDNA using the first-strand cDNA synthesis kit (AT311, TransGen Biotech, Beijing, China). Primers for qRT-PCR were designed by Primer 5.0, and α-tubulin (*TUA*) was used as an internal reference gene [54]. These primers are displayed in Table S6. qRT-PCR was carried out under the StepOne Plus program (Applied Biosystems, Foster City, CA, USA) using SYBR Green Real-time PCR Master Mix (QPK-201, Toyobo Bio-Technology, Shanghai, China). Each PCR mixture was a 10 μL system that contained 5 μL of SYBR Green Real-time PCR Master Mix, 1 μL of 20-fold diluted cDNA, 3.2 μL of sterile water, and 0.4 μL of each primer. The amplification procedure was as follows: predenaturation at 95 °C for 2 min, denaturation at 95 °C for 10 s, annealing and extension at 60 °C for 30 s; this process was repeated 40 times. The remaining procedures were the program default parameters. Each reaction was detected with three independent replicates. The relative expression levels of *PmTCP* genes were computed according to the 2^−ΔΔCT^ method [55]. One-way ANOVA and multiple comparison tests were analyzed by GraphPad Prism 8.0 software.

### 4.8. Transcription Self-Activation Detection

The open reading frame (ORF) of the *PmTCP7* gene was fused with the pGBKT7 vector cut by EcoRI and BamHI double enzymes. The primers for vector construction are provided in Table S5. Then, the correctly sequenced recombinant vector was transformed into AH109 yeast strains (YC1010, Weidi Biotech, Shanghai, China), which were subsequently cultured on the SD/-Trp medium at 28 °C for 3 d. Single yeast colonies were selected for PCR validation, the successfully transformed yeast single colonies were collected in SD/-Trp liquid medium, and 5 μL of diluent was inoculated onto the surface of yeast medium SD/-Trp and SD/-Trp/-Ade/-His, respectively. Meanwhile, 5 μL of diluent was inoculated on the SD/-Trp/-Ade/-His solid medium containing X-α-gal. Subsequently, the growth status of yeast cells was observed and recorded.

## 5. Conclusions

In this study, we identified 13 PmTCP TFs from transcriptomes of *P. massoniana*. These PmTCPs were divided into Class I and Class II categories, and different subgroup members had different motif compositions. Members of the same subgroup had similar conserved motif composition, indicating that they may perform similar functions. According to the transcriptome of drought stress, seven *PmTCPs* were found to be responsive to drought stress. Meanwhile, the expression patterns of the selected four *PmTCP* genes under different conditions suggested that they may have important functions on several stress responses and play multiple roles in different stages of plant growth and development. These findings provide a theoretical basis for the study of TCP TFs, are conducive to further understanding the functions of PmTCPs, supply potential strategies for breeding in *P. massoniana*, and contribute to the study of the mechanism resistance ability of other plants.

## Data Availability

The data presented in this study are available in Supplementary Materials.

## Supplementary Materials

The following supporting information can be downloaded at https://www.mdpi.com/article/10.3390/ijms242115938/s1.
